# A Panel Management Approach Using Prescription Drug Monitoring Program Data for Primary Care Patients with Chronic Pain Treated with Opioids: A Feasibility Study

**DOI:** 10.21203/rs.3.rs-7793877/v1

**Published:** 2025-10-08

**Authors:** Constance van Eeghen, Marianne Burke, Zoe Daudier, Amanda G. Kennedy, Neil Korsen, Benjamin Littenberg, Moira Mulligan, Doug Pomeroy, Jennifer Raymond, Meagan E. Stabler, Charles D. MacLean

**Affiliations:** University of Vermont; University of Vermont; Dartmouth Health; University of Vermont; MaineHealth; University of Vermont; University of Vermont; University of Vermont; Dartmouth Health; Dartmouth Health; University of Vermont

**Keywords:** Primary Care, chronic pain, opioid, drug prescription, panel management

## Abstract

**Background:**

This feasibility study explored a process for primary care clinicians to improve chronic pain management related to opioid prescribing practices by using state-based Prescription Drug Monitoring Program (PDMP) data to create panel management reports on patients receiving long term opioid therapy.

**Methods:**

Conducted across four rural primary care clinics in Northern New England, the study assessed the feasibility of downloading and utilizing PDMP data and the perceived value of panel management reports derived from both PDMP and electronic health record (EHR) data in the care of patients with chronic pain treated with opioids for more than one year.

**Results:**

The study found that downloading PDMP data was feasible and efficient across all sites. However, EHR review proved more challenging due to inconsistencies in data entry and the unstructured nature of some relevant data fields. Clinicians generally found PDMP data easy to generate and the panel management reports informative and useful for understanding opioid prescribing trends and identifying high-risk patients.

**Conclusions:**

The findings suggest that while PDMPs are a potential source for panel management reports for patients with chronic pain who are treated with opioids, further study is needed to determine the effectiveness of such efforts to improve care for and safety of patients treated with opioids.

## Background

The most common symptom encountered in primary care is pain,^1^ making primary care clinicians (PCCs) the *de facto* pain management service in the U.S. With PCCs writing half of all opioid prescriptions and writing a higher proportion of long-term opioid prescriptions,^2, 3^ they are the primary managers of a treatment that may lead to opioid use disorder (OUD) and its associated complications, including death. For 30 years, the U.S. population has experienced increased opioid use partly due to prescribed opioids. Although opioid prescribing has declined since 2010,^4^ opioid-related deaths have increased in the U.S to 32.6 deaths per 100,000 standard population in 2022, recently decreasing to 31.3 in 2023^5^. The 29% annual increase of deaths from 2020–2021 was likely due to the availability of illicit synthetic opioids (e.g., fentanyl^6^), lack of compliance by insurers in coverage of care for substance use disorder, barriers to accessing evidence-based treatment, and other secular trends.^7^ Opioid prescribing continues to be of interest as current trends show primary care physicians are decreasing opioid prescribing at a decreasing rate (leveling off) while advanced practice primary care clinicians are increasing their prescribing.^8^

Treating chronic pain with opioids is challenging. Long-term prescribing is associated with many patient characteristics, including age at initiation (adults aged 18–25 years according to some sources; older adults in others), low socioeconomic status, and poor physical and mental health.^9,10^ Predictors of prescription opioid misuse for patients with chronic pain are complex, involving multiple psychosocial and mental health factors.^11^ In 2021, 3.1% of the population aged 12 years or older (almost 9 million people) reported that they misused prescription pain relievers (primarily but not solely opioids).^12^ Most often, they sought relief from physical pain.

Healthcare clinicians are caught between under-addressing patients’ pain and over-prescribing a potentially addictive substance. Even a five-day course of opioids is associated with a 10% probability of long-term opioid use one year later,^13^ rising to 27.3% for long-acting opioids. Tramadol, promoted as safer than traditional opioids in avoiding addiction, is associated with a one-year probability of long-term use of 13.7%.^11^ Although national guidance exists for opioid treatment of chronic, non-cancer pain,^14^ there is evidence of divergence between prescribing practice and clinical recommendations for a variety of reasons,^15^ including “inherited” patient panels from retiring PCC colleagues.^16^

PCCs need data and tools to manage chronic pain appropriately, including opioid prescribing. They must optimize patient outcomes while also following prescribing guidelines, state and local regulations, insurance requirements, and institutional policies. One strategy is “panel management,” which offers a systematic approach to the management of chronic illness.^17–19^

Panel management is useful in managing chronic conditions such as diabetes mellitus and hypertension.^18^ It is characterized by a set of tools and processes to identify patients and provide structured workflows based on evidence-based protocols, especially for those at high risk. Well-established models exist and they highlight the importance of using population health approaches such as the development of chronic disease registries.^20^ However, the model depends on customizable, timely data reporting under local control and the ability to benchmark to guide improvement.^21^

One possible information source for panel management of patients treated with opioids for chronic pain is state-based Prescription Drug Monitoring Programs (PDMPs) that track controlled substance prescriptions. PDMPs feature data downloads for longitudinal quality improvement (QI) and reporting purposes, although individual prescribers can see data only of patients for whom they prescribe. Such data, when aggregated across clinicians, can provide a practice-wide view of prescribed medications, dosage, prescribing intervals, and concurrent prescriptions of benzodiazepines, which can inform a panel management approach to opioid treatment.

We conducted a feasibility study across four primary care clinics in Northern New England (NNE) to assess whether: 1) PCCs and staff could download, merge, and deidentify PDMP data for analysis and 2) PDMP and electronic health record (EHR) panel management reports on opioid prescribing were considered valuable by PCCs.

## Materials and Methods

### Study Design

This retrospective, observational, feasibility study assessed PCC and staff ability and willingness to collect patient data from the PDMP for primary care practices in three different states and to match a sample of those records with the EHR for chart review. Practices collected PDMP data from the previous 3–6 years (2017–2022) as allowed by individual state PDMP regulations. The downloaded data from each prescriber were combined into a single, de-identified dataset by the site staff to create a complete prescribing history of each patient in the practice. Trained practice staff also extracted EHR data from the previous two years. The research team sought to assess 1) whether these sources provided insights into opioid prescribing patterns and 2) adherence with state-level prescribing regulations and Centers for Disease Control and Prevention (CDC) prescribing guidelines. The research team analyzed deidentified data and generated prescriber- and practice-specific longitudinal trend reports on populations of patients with chronic pain treated with long-term opioids — the specific panel of interest. Prescribers from each clinic reviewed the reports and participated in a structured group conversation about their perspectives on the ease of collecting the data and the usability of the reports.

To help guide the work of this study, the research team convened a team of patients and clinicians to advise on a data collection instrument for chart abstraction to include indicators of pain management relevant to the population. The team included two patients with long-term chronic pain and opioid treatment experience, a clinical pharmacist, and a PCC. Patient partners were compensated for their time and contributed to instrument design, interpretation of results, and dissemination efforts. Meeting ten times over this one-year project, the patient/clinician partner team reviewed the chart abstraction data collection instrument and advised on key features of pain and pain management to identify during chart review. They evaluated available patient-reported instruments, highlighted strengths and gaps, and made recommendations for future work on this topic. They collaborated on the development, editing, and review of a poster and an oral presentation on the results of this study.

### Study Participants/Population

Eligible primary care clinics were recruited through the Northern New England CO-OP Practice- and Community-Based Research Network. Each eligible clinic had at least 50 patients across all clinicians that were treated for chronic pain with opioids for at least 90 days. The subjects of analysis were adult patients (18 years+) with chronic pain treated with 1000 or more morphine milligram equivalents^22^ (MME) of opioids per year, or 5 or more individual opioid prescriptions in a year, between 2017 and 2022. Patients who were receiving buprenorphine or other medications for OUD were excluded. Patients receiving buprenorphine for chronic pain were included.

### Procedures, Materials, and Instruments

Clinics completed an initial questionnaire to describe their office practice, patient panel size, number and types of clinicians, and typical pain management and assessment tools used with patients (Appendix 1).

#### PDMP Downloads and Data Merge:

The research team trained prescribers to download their prescribing history from the PDMP using a written manual and a 10-minute video guide with step-by-step procedures developed for this study (Appendix 2, Part 1). A designated practice staff member combined and deidentified the data into a single dataset to create a complete prescribing history of each patient in the practice (Appendix 2, Part 2). Each practice dataset was delivered to the research team through secure file transfer. From this file, the research team created a roster of 50 patients treated with long-term opioids (LTO Roster) for each practice, assuring proportional representation of each prescriber. LTO Roster records were coded to match patient identifiers held only by the practice behind its organizational firewall.

#### EHR Chart Review:

The research team trained clinic staff in EHR chart review, using a written manual (Appendix 3) to accompany the REDCap^23^ data entry abstraction instrument (Appendix 4). The team conducted live remote training sessions for two clinic staff members selected by each practice. As part of each training session, chart reviewers practiced reviewing one EHR record matched to the LTO Roster working together and one record separately, cross-comparing results, and discussing any differences. Separate coding of additional records continued until the two reviewers coded the same record consistently, after which they continued independently to complete their review of the remaining patients on the LTO Roster. Reviewers abstracted de-identified EHR data into REDCap, capturing a two-year look-back period prior to the patient’s most recent chronic pain-related visit in 2022.

#### Practice Presentations and Guided Group Discussion:

The research team conducted an hour-long presentation and guided discussion session with each practice following the analysis of practice data. The meetings included the prescribers and the practice manager with the research team. A presentation described the controlled substance prescribing trends at the practice, including variation among prescribers in the practice and comparison to other practices in the study. The study team used a semi-structured small group discussion guide to gather the perspective of the participants on the ease of data retrieval and the usefulness of the reports as predictors of acceptability (Appendix 5).

### Study Measures and Analyses

#### PDMP Data:

Each practice dataset provided a de-identified patient number assigned by the practice, year of birth, prescriber identifier, date of prescription, drug name, number of days supplied, and dosing in MMEs. Other than the birth year, no other demographic patient characteristics are provided by the PDMP. The research team summarized the data at the prescriber and practice level. Descriptive statistics included annual sums of opioid MMEs prescribed, number and proportion of high-risk patients (i.e., greater than 90 MME/day or overlap of opioids with benzodiazepines), proportion of prescriptions in multiples of 7-days (to assure that prescriptions are consistently due on a weekday to avoid on-call coverage), and number of patients prescribed with both an opioid and benzodiazepine. Panel management reports included tables and charts highlighting trends in prescribing practices over time.

#### EHR Data:

The research team used descriptive statistics to analyze data reported from chart reviews, including the proportion of patients with documentation of legally mandated procedures (use of the PDMP, informed consent, treatment agreement) and CDC-recommended measures (pain, opioid risk, functional status, urine drug testing, and screening for depression). Quantitative PDMP and EHR analyses were conducted using Excel and Stata version 18 (StataCorp, College Station, TX).

#### Feasibility, Ease of Use, and Usefulness:

The research team assessed the feasibility of conducting PDMP downloads and extraction from EHRs in real time as participants engaged in the study. The assessment was based on ease of use of the data retrieval process and usefulness of the panel management reports as stated in responses given in the guided discussion sessions after each practice had reviewed their results. After each session, the research team met to review the session responses, organize the qualitative data by practice and theme, and come to consensus on what was learned.

### Protection of Human Rights and Participation Incentives

The Institutional Review Boards at the University of Vermont and the Dartmouth-Hitchcock Medical Center determined that the study was not human subjects research per the regulatory definition under 45 CFR 46.102(d) and that a full review was not needed. Business associate agreements and memoranda of understanding were completed by clinics and the research institution (University of Vermont). Clinics received a stipend for participation and for compensation of the staff who reviewed the EHR, as well as a report on their outcomes in comparison with other study participants.

## Results

Four primary care practices in rural areas of Maine (1 clinic), New Hampshire (1), and Vermont (2) participated. The ownership model, size, and other practice characteristics are shown in Table 1. Between 4% and 12% of patients used opioids during the observation period available in the PDMP. Patients in the EHR review study sample (48–49 patients per practice successfully matched to the PDMP registry) were more likely to be female, except for site D. The median age ranged from 58–70 (Table 2).

### 1. Overall Feasibility

All practices reported that the PDMP download and data collation procedures were feasible and efficient, completing them successfully. Additional training and help were needed in one practice for the de-identification step (Appendix 2, Part 2).

Feasibility of the EHR review was not as positive. Although each site was able to complete the chart review process, training the chart reviewers required multiple clarifications and re-training for each practice. Locating the relevant EHR data endpoints was consistently more successful when associated with a structured or mandatory field in the EHR (e.g., report of PDMP lookup or informed consent and treatment agreements). When reviewers looked for non-standardized data that might be found in multiple EHR fields or record sections, such as functional assessments or discussions of non-medical treatments, their ability to find the data was less consistent and reliable.

### 2. Value of PDMP Data: Ease of Use and Usefulness

**Figure 1:**
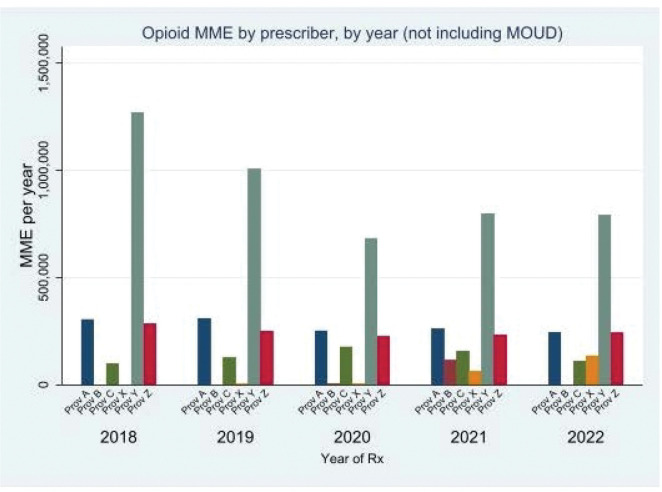
Example graphic of trend in opioid prescribing for pain in MME, by anonymized prescriber, 2018–2022 Example of Trend in Opioid MME by prescriber for a partial, anonymized practice. This bar chart is an example of a trended report of opioid prescribing for pain that was included in the wrap-up session with the practice. Each bar represents an individual primary care provider, with bar height displaying the total MMEs prescribed for each of the years 2018–2022. Note the relevance of on-site context when interpreting these charts: the change in MME/year for Provider Y may be due to the gradual adjustment of opioid treatment for a panel of patients “inherited” from a recently retired prescriber. MOUD: Medication for Opioid Use Disorder

**Figure 2:**
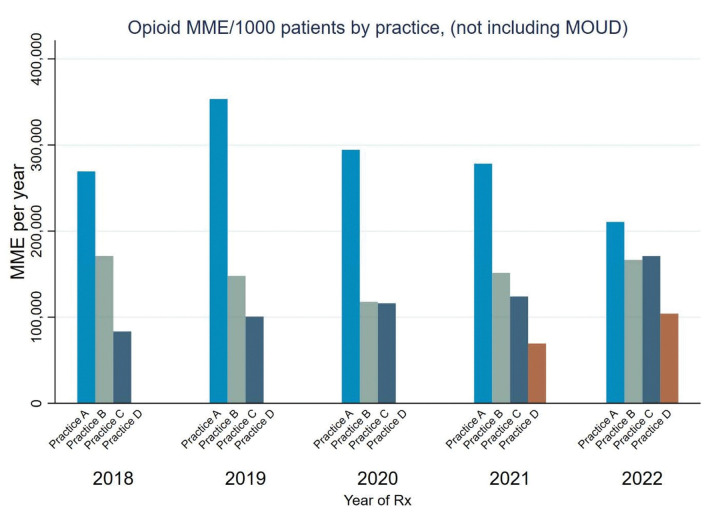
Example graphic of trend in opioid prescribing for pain in MME, by anonymized primary care practice, 2018–2022 Annual opioid MME prescribed by the practice per 1000 patients in the practice panel. Trends of opioid MME prescribed by clinicians at each participating clinic varied based on the particular histories involved in retiring and newly arrived prescribers and greater awareness of opioid prescribing strategies and monitoring tools. Opioid prescribing per 1000 patients, by 2022, appeared to have converged between 100K-200K MMEs/year. MOUD: Medication for Opioid Use Disorder

Panel management reports from the PDMP about opioid prescribing for patients with chronic pain were presented in-person to clinicians at each site in guided group discussions. These were made available in tabular (Table 3, Table 4) and bar chart formats (Fig. 1, Fig. 2). Clinicians described the reports as generally informative and useful, even when summary reports from other sources were available to them. Several clinicians noted that the panel management reports provided novel insights into variation across prescribers in the practice and across time. Some highlighted that the reports provided actionable information for QI and managing patient care.

Prescribers and office managers reported that the challenges of opioid prescribing for chronic pain continue to be a high priority and that new strategies for managing care are needed. In response to “Is Panel Management report information useful to your practice?,” two sites agreed with “Yes” and two sites with “Maybe,” with one prescriber noting, *“Maybe for some kinds of patients… It’s hard to work it in in the time with patients and keep it a priority”* (Table 5). The value of these reports was also reflected in comments such as “*We want to be on the same page*” in prescribing opioids for pain and caring for their community and “*patients are aging over time and their past treatment plans, in the context of the whole picture of their health, are no longer working*” (Table 5). The panel management reports demonstrated one method of providing the information needed to achieve these goals. Although three practices reported that these data were available in their EHR or PDMP vendor sources, they were not considered flexible in format or actionable for follow-up and they did not provide a practice-level view of prescribing.

### 3. Value of EHR Data: Ease of Use and Usefulness

Clinicians reported that the chart review reports were useful and accurate but not novel. The information usually could be provided through their clinical systems although such reports were not easily available or well organized. Presence of patient-centered measures (e.g., pain, function, and risk stratification) in the EHR review varied within and across practices (Table 2).

Legally mandated endpoints such as patient treatment agreements and PDMP lookup dates were well documented. Informed consents were often not found, likely because such documentation for a long-term condition would fall outside the 2-year window of the chart review. Discretionary strategies, such as pill counts, pain measures, functional assessments, screening for depression, OUD risk assessments, and offering or discussing non-medical therapy were typically lower and varied widely. In Table 2, clinicians were given credit for documentation of functional status for use of either a scale such as the “Pain, Enjoyment, and General Activity” (PEG) assessment, or for any non-standardized assessment reflected in the clinic notes. Measures that were structured into designated data fields (e.g., depression screen) were consistently documented (76%-98% across clinics); measures that had no standardized documentation field (functional status) were not (2%-60%). Clinicians noted that consistent use of these tools would be beneficial and regular reports “*would push us to standardize [our] practice*” (Table 5).

### 4. Challenges identified

The research team encountered specific challenges in training and supporting practice sites with the data download and chart review requirements. Each state governs the access to and use of PDMPs and may have different restrictions on how long historical data are available, requiring regular downloads to update trend reports in keeping with prescribers’ need to review panel management reports. In addition, healthcare organizations have differing requirements on how their clinical data may be used for QI projects, such as multiple levels of review and approval. Creating precise but not overly laborious instruction for EHR abstraction was an ongoing goal. Even with better instructional materials, however, chart review remained a time-consuming process.

## Conclusions

This study demonstrates that it is feasible to create reports from PDMP downloads for PCCs to use in caring for patients with chronic pain. PCCs found the process easy and the results useful, but not always novel with respect to past reports some had received. However, clinicians found the format of panel management reports valuable.

With opioid prescribing largely now in the domain of primary care^3, 24^, PCCs can leverage panel management strategies that are already familiar to them. The CDC chronic opioid prescribing guidelines of 2016 and 2022 highlighted the importance of specific strategies to support safe and responsible prescribing^14^. For busy clinical teams, access to summary data, peer comparisons, and rosters of patients around which to plan QI programming can be very useful. In this pilot study we demonstrated the feasibility and usefulness of using a widely available data source, the state PDMP, to create helpful data summaries.

While EHR data represent medications that were prescribed, PDMP data reflect medications that were actually dispensed at any pharmacy in the state (and often other states as well). There may be important discrepancies between prescribed and dispensed medications. For example, in post-operative outpatient prescribing, while 92% of patients receive an opioid prescription, only 27% of the prescribed MME was consumed^25^. Summary reports available through EHR systems vary not only by vendor, but also by specific institutional customization—this limits the ability to compare data reports across practices. When compared to PDMP vendor reports (which are limited to single clinicians), this approach to panel management reports can provide a practice-level view of the care management of patients with chronic pain treated with opioids and can avoid the problem of double counting patients who receive prescriptions from more than one prescriber. Despite these advantages, reviewing data on opioid prescribing alone does not provide a complete clinical picture. An understanding of the particular context within a practice is essential to account, for example, for prescribers who have recently retired, new clinicians managing legacy patient panels, or prescriber specialization (pain management, opioid use disorder, end of life care, etc.) when interpreting the data.

In an effort to create a more complete picture of opioid prescribing in primary care, we also sought to extract patient-reported outcomes from the EHR. Recent reports have highlighted that many patients with chronic pain treated with opioids are interested in reducing their dosage, but are concerned about being able to control their pain, mood, and opioid cravings effectively^26^. The inclusion of patient-reported outcomes on pain management and tolerance is a key part of treatment, as no objective measure of pain exists. In this small sample of four primary care practices in one region of the US, we observed considerable variability in the documentation of pain, functionality, and risk assessment in the EHR. This raises the question of whether a standardized assessment tool that incorporates administrative endpoints (such as those that are legally required) and important patient-reported outcomes (such as pain, function, and perspective on opioid tapering) would be helpful in standardizing care and benchmarking. Reducing opioid doses increases the chance that the patient experience will worsen, although this is not always true^27^. Clinicians and researchers have an obligation to measure the impact of changes in pain management regimens on those they care for, the patients.

Strategies to support PCC efforts to improve opioid medication management already indicate the value of identifying and tracking the use of opioids. EHRs cannot generate rosters based on medication dispensing across the state and by multiple prescribers. Those depending on standard PDMP reports will not see trends at the practice level. And those who can conduct manual chart abstraction will face the inefficiencies of unstructured data as well as lacking state-wide information available from pharmacies dispensing Schedule II-IV controlled substances. The use of clinical data to create panel management reports is not new, but the use of clinical data from outside the patient record for panel management may provide a new opportunity to work with patients on the challenges brought by opioid treatment. The PDMP also provides a highly accurate and widely available source of data for research on prescribing patterns of controlled substances.

Limitations to this study include uncertain generalizability beyond the four clinics that volunteered to participate, all of whom were in rural, northern New England settings. Further work is needed to assess feasibility across a geographically wider set of clinics with varying needs related to opioid prescription management. Small group, semi-structured discussions elicited feedback from participants willing to voice their opinions in front of their peers and may have been influenced by social desirability bias. Combining the prescribing history of 3–6 years of PDMP data with two years of abstracted EHR data may have created an incomplete picture of compliance with state-level regulations and CDC guidelines. We may have missed documented care that stood just outside the 2-year time horizon of the chart review.

Some patients who begin opioid treatment may be lost to follow-up in the PDMP data base due to a change in state of residence, movement to a long-term care or correctional facility, death, or discontinuation of opioid treatment. Use of the PDMP as a source for panel management should include watching for the absence of patients on the population roster and including a follow-up mechanism for those who are missing.

In summary, this feasibility study across four primary care clinics in northern New England revealed that while downloading PDMP data was feasible, easy, and efficient, EHR chart review was more challenging due to inconsistencies in data entry. Clinicians found panel management-style reports for patients with chronic pain treated with opioids to be informative and useful. PDMPs may be a helpful source for a panel management approach in caring for patients with chronic pain and treated with opioids, but further research to test its comparative effectiveness, its impact on primary care clinicians of different locations and credentials, and the value of more standardized applications of clinical data across state-based PDMPs and EHRs is needed.

## Supplementary Material

Supplementary Files

This is a list of supplementary files associated with this preprint. Click to download.

• AppendicesTOCofTrainingMaterialsandDatacollectionInstruments.pdf

• App1PraciticeInfoQuestionnaire.pdf

• App2P1PDMPDownloadData.pdf

• App2P2PDMPMergeDeidentifySOP.pdf

• App3ChartreviewSOP81723.pdf

• App4ChartReviewsurveyOpioidPrescrREDCap4292023.pdf

• App5Postpresentationsemistructuredguide.pdf

## Figures and Tables

**Table 1:** Practice characteristics

Practice ID and location	A: State 1	B: State 1	C: State 2	D: State 3
Practice ownership model	CHC/FQHC *	CHC/FQHC	Health system	Health System
Specialty	FM ^†^	FM	Mixed FM & IM^‡^	FM
Tax status	Not-for-profit	Not-for-profit	Not-for-profit	Not-for-profit
NCQA - PCMH status ^§^	Participating	Participating	Participating	Participating
Electronic health record vendor	Athena	Medent	EPIC	EPIC
Year EHR installed	2015	2017	2013	2011
Clinicians in practice	7	8	13	11^a^
Patients in practice	5,575	11,465	9,992	8,263
Patients visits per year	3,791	9,909	7,721	6,301
Years of observation in PDMP	6	6	6	3
Number of patients using an opioid in PDMP dataset (%)	636 (11%)	1,378 (12%)	1,170 (12%)	313 (4%)
Age of patients in PDMP, mean (SD)	69 (16.1)	63 (15.6)	64 (16.0)	63 (14.6)
Insurance mix				
Percent panel with Medicare	24%	28%	IM 35%, FM 19%	23%
Percent panel with Medicaid	missing	24%	missing	5%
Patient-facing tools				
Depression screening	PHQ ^‖^	PHQ	PHQ	PHQ
Initial misuse risk assessment	ORT^¶^	None	ORT, CPAA^#^	ORT
Ongoing risk assessment	None	None	None	None
Informed consent and treatment agreement	Yes	Yes	Yes	Yes
Pain/functional assessment	Custom	PEG **	PEG	PEG
Non-medical roles in practice				
Psychologist	Yes	-	-	-
Social work	Yes	-	Yes	Yes
Pharmacist	-	-	Yes	Yes
Psychiatry APRN ^††^	Yes	-	-	-
MOUD team^‡‡^	Yes	-	-	-
Community Health Worker	Yes	-	-	Yes
Health Coach	-	-	Yes	-
Behavioral Health partner or consulting psychiatrist	-	Yes		Yes
Nurse care coordinator	-	-	-	Yes

* CHC/FQHC Community Health Center/Federally Qualified Health Center

† FM Family Medicine

‡ IM Internal Medicine

§ NCQA - PCMH status

‖ PHQ Patient Health Questionnaire

¶ Opioid Risk Tool

# Chronic Pain Assessment Algorithm

** Pain Enjoyment General activity scale

†† Advanced Practice Nurse Practitioner

‡‡ MOUD Medication for Opioid Use Disorder

a 11 of 23 prescribers in Site D participated in the study. All other practice characteristics reportedfor Site D represent the entire practice.

**Table 2:** Medical record documentation of best-practice endpoints

Practice ID and location	A: State 1	B: State 1	C: State 2	D: State 3
Number of records reviewed	49	49	48	49
Age, median (range)	70 (18–90+)	59 (18–90+)	58 (18–90+)	66 (34–90+)
Female, proportion	59%	55%	53%	39%
Legally mandated endpoints				
PDMP lookup documented *	94%	72%	100%	90%
Treatment agreement	94%	94%	98%	62%
CDC-recommended strategies				
Informed Consent	61% in 2 yrs	18% in 2 yrs	42% in 2 yrs	67% in 2yrs
Pill count	12%	12%	2%	12%
Urine drug testing	92%	56%	98%	92%
Pain measurement	65%	Not found	100%	18%
Functional status ^†^	57%	60%	2%	41%
Depression screen	98%	76%	97%	88%
OUD risk assessment, (tool) ^‡^	Not found	Not found	21% (ORT ^§^)	33% (ORT)
Documentation of non-opioid treatments, proportion	53%	44%	40%	59%

* PDMP: Prescription Drug Monitoring Program

† Assessed with PEG or in clinician notes

‡ OUD: Opioid Use Disorder

§ ORT: Opioid Risk Tool

**Table 3:** Example of trend in opioid prescribing for pain in MME, by anonymized prescriber, 2018–2022

Prescriber	Year					% change MME (18–22)
	2018	2019	2020	2021	2022	
Clinician A	305,416	310,398	252,561	261,959	245,404	−20%
Clinician B			4,883	115,775		-
Clinician C	99,240	125,679	177,528	157,548	111,174	+ 12%
[Partial practice data shown for anonymity]						
Clinician Y		1,158	4,655	63,156	136,104	-
Clinician Z	1,271,132	1,007,300	683,920	799,567	792,123	−38%
Practice Total *	1,961,865	1,695,875	1,348,993	1,631,280	1,526,990	−22%

* Totals may not sum because of partial practice data shown

**Table 4:** Example Patient counts by anonymized prescriber, 2018–2022

Prescriber	2018	2019	2020	2021	2022
Clinician A					
Count of opioid patients	151	103	112	91	80
Count of chronic opioid patients	49	36	33	33	32
Proportion 7 pill increments	36%	46%	47%	47%	49%
Count of benzo patients	76	65	51	60	51
Count of overlap patients	16	14	16	16	13
Count of MOUD* patients	0	0	0	21	17
Clinician B					
Count of opioid patients			8	62	
Count of chronic opioid patients			1	22	
Proportion 7 pill increments			36%	36%	
Count of benzo patients			6	43	
Count of overlap patients			1	12	
Count of MOUD patients			0	0	
Clinician C					
Count of opioid patients	46	56	55	43	38
Count of chronic opioid patients	15	22	21	22	21
Proportion 7 pill increments	66%	76%	77%	74%	79%
Count of benzo patients	74	74	70	69	56
Count of overlap patients	17	18	9	12	10
Count of MOUD patients	0	0	0	0	0

* MOUD Medication for Opioid Use Disorder

**Table 5:** Qualitative Responses to Semi-Structured Guided Group Discussions

	A State 1	B State 1	C State 2	D State 3
Number of attendees	7	9	8	16
Questions/Themes				
Is PDMP download easy?	Easy; not even memorable	Yes; [QI leader] came by and coached each clinician through it; easy	Mostly	Yes; Took maybe 5 seconds; would be even easier if made into a routine
Are PDMP data useful?	Not anymore. Have been working on this 5–10 years; concentrated efforts to reduce opioid prescribing. Other aspects are more challenging: decreasing high doses	Confirms what is known. [This report] added new info (overlap patients) which is useful and not currently reported.	Yes. Is it actionable? What are the guidelines we should use; what project should we do? Community is changing from mill town to “up and coming.” How does this show up in the data? Are there better ways to look at the data? We want to be on the same page as a practice and a community. Consistent approach is important. Data reflects the community.	Yes. Interesting; feel for aggregate number is good. Important to separate the 2 populations: pain management and MOUD; would be very useful with an Opioid Council
Do you already have PDMP data available?	Previously available in annual reviews. QI projects with similar data: UDS, contract, agreement	Most of it. Clinicians are very aware of their opioid prescribing and patients treated with opioids already.	Not known or accessible or easy to get to. There are some Best Practice Alerts on multiple drugs and Z drugs; possibly a high risk elderly warning exists somewhere.	No. Technically possible but not operationalized; only data they see comes from financial DB
Are chart review data useful?	Confirms what is known. [We] get information through clinical system	Confirms what is known	Yes. Can get information through clinical system. Have EMR flags based on pt med list	Yes. Data would push us to standardized practice
Is Panel Management report information available from your own system?	Yes	Yes	Yes. This report is better	No
Is Panel Management report information useful to your practice	Maybe for some kinds of patients. High dose patients “age out” - they die. QI is a challenge. Lots of guidance is available. It’s hard to work it in in the time with patients and keep it a priority.	Maybe, if it is actionable. There is a sense that patients are aging over time and that past treatment plans, in the context of the whole picture of their health, are no longer working, including opioid prescribing. Their quality of life is important and their opioid prescription is part of that.	Yes. High prescribers took over retiring practices and [have] many opioid-using patients. [We need to be] making practice structured.	Yes. Data would push us to standardized practice.

## Data Availability

Due to the sensitive nature of the data shared by health care clinicians for this study, and based on their respective state laws governing use of prescription drug monitoring data, we assured participants that their raw data would remain confidential and not be shared. Data are not available. The data that have been used are confidential. Open materials statement: the components of the research methodology needed to reproduce the reported procedure(s) and analyses are not publicly available but available on request to the corresponding author.

## Abbreviations

CDC: Centers for Disease Control and Prevention
HER: Electronic Health Record
LTO: Long-Term Opioids
MME: Morphine Milligram Equivalents
NNE: Northern New England
ORT: Opioid Risk Tool assessment
OUD: Opioid Use Disorder
PCC: Primary Care Clinician
PEG: Pain, Enjoyment, and General Activity assessment
PDMP: Prescription Drug Monitoring Program
